# A randomized trial of mailed questionnaires versus telephone interviews: Response patterns in a survey

**DOI:** 10.1186/1471-2288-7-27

**Published:** 2007-06-26

**Authors:** Helene Feveile, Ole Olsen, Annie Hogh

**Affiliations:** 1 National Research Centre for the Working Environment, Copenhagen, Denmark

## Abstract

**Background:**

Data for health surveys are often collected using either mailed questionnaires, telephone interviews or a combination. Mode of data collection can affect the propensity to refuse to respond and result in different patterns of responses. The objective of this paper is to examine and quantify effects of mode of data collection in health surveys.

**Methods:**

A stratified sample of 4,000 adults residing in Denmark was randomised to mailed questionnaires or computer-assisted telephone interviews. 45 health-related items were analyzed; four concerning behaviour and 41 concerning self assessment. Odds ratios for more positive answers and more frequent use of extreme response categories (both positive and negative) among telephone respondents compared to questionnaire respondents were estimated. Tests were Bonferroni corrected.

**Results:**

For the four health behaviour items there were no significant differences in the response patterns. For 32 of the 41 health self assessment items the response pattern was statistically significantly different and extreme response categories were used more frequently among telephone respondents (Median estimated odds ratio: 1.67). For a majority of these mode sensitive items (26/32), a more positive reporting was observed among telephone respondents (Median estimated odds ratio: 1.73). The overall response rate was similar among persons randomly assigned to questionnaires (58.1%) and to telephone interviews (56.2%). A differential nonresponse bias for age and gender was observed. The rate of missing responses was higher for questionnaires (0.73 – 6.00%) than for telephone interviews (0 – 0.51%). The "don't know" option was used more often by mail respondents (10 – 24%) than by telephone respondents (2 – 4%).

**Conclusion:**

The mode of data collection affects the reporting of self assessed health items substantially. In epidemiological studies, the method effect may be as large as the effects under investigation. Caution is needed when comparing prevalences across surveys or when studying time trends.

## Background

Mailed questionnaires and telephone interviews are both cost-effective methods of data collection[1] and combined they may reduce non-response [2-5]. Pooling of the two types of data requires caution if different modes of data collection elicit different patterns of responses or obtain responses from different subsets of the population. Also comparisons between studies or over time may be biased if different modes of data collection have been used in the studies.

No systematic reviews on the topic were identified in Medline or Cochrane Library. However, we identified several studies which inspired us to the following hypotheses:

1. Telephone interviews result in higher response rates than mailed questionnaires[2,6-8]

2. The rate of missing responses is higher among questionnaire respondents than among telephone respondents[4,7-9]

3. Willingness to answer sensitive questions and questions concerning socially undesirable behaviour is greater in questionnaires than in telephone interviews[1,7,8]

4. Telephone respondents have a preference for extreme response categories compared to questionnaire respondents[7,8]

5. Telephone respondents report better health and well-being than questionnaire respondents[1,4,7-10].

The aim of the present paper is to provide quantitative estimates of the effects of data collection modes in epidemiologic studies. The paper is based on a Danish national survey of psychosocial work environment, health and well-being[11] in which 35% of the sample was randomised to telephone interview and 65% to receive a mailed questionnaire. Even though the primary purpose of the survey was developing a questionnaire measuring aspects of the psychosocial work environment, a sample of adults were contacted irrespectively of their labour market status. This approach ensured a national representative sample. Those presently working were asked multiple questions about their psychosocial work environment, but all respondents were asked questions concerning health and well-being, permitting validation of health and well-being scales.

## Methods

The Danish, centralised civil register (CRS) contains information for every person who is or has been an inhabitant of Denmark since 1968 and is updated daily[12]. Adults aged 20–60 registered in the CRS were eligible. The sample size was 4,000 persons (slightly larger than the previously published trials[1,2,4]). A stratified, systematic sample of all adults aged 20–60 residing in Denmark was drawn from the CRS. Stratification was done according to gender and year of birth. For each gender 50 persons were sampled from the years 1938–1976 (with the following odd exceptions: 39 women born in 1972, 61 women born in 1973, 40 men born in 1972 and 60 men born in 1973). From the limiting years 1937 and 1977, 25 men and 25 women were sampled.

### Assignment

The allocation sequence for the randomization was generated by a computer assistant using the SAS routine RANUNI and a subsequent sorting procedure. The allocation lists were delivered to the head of the project.

### Masking

Neither participants, nor surveyors or assessors were blind to group assignment.

Those randomised to receive a mailed questionnaire were sent one at their current address. Non-respondents received two postal reminders at intervals of three weeks. The second reminder included a questionnaire. For the group randomised to telephone contact, phone numbers were searched electronically in the database of the national phone company and manually in additional databases. Persons with a confidential phone number were sent a letter with an invitation to call back. The telephone interviews were computer assisted; they were preceded by a letter and were primarily conducted Monday-Thursday between 4 pm and 9 pm and Sunday between noon and 4 pm. The interviewers were student assistants and paid by the hour. They received a short course, a written manual and conducted three test interviews. The interviewers were instructed to accept item refusals and act neutral, but whether they complied completely with these instructions is uncertain, just as it is uncertain whether all response categories consistently were read out. Non-respondents were followed up with at least three calls at different times of the day/evening. Those unwilling to participate in a telephone interview were offered a mailed questionnaire. The latter are treated as non-respondents here. In both of the randomised groups, the study was presented as concerning health, well-being and, for those having a job, the psychosocial work environment. No incentives were used in either of the two randomised groups.

The questionnaire was 27 pages long and contained 257 items. The duration of telephone interviews was typically 30–45 minutes (cf. English translation[13]). This paper deals with the 47 items concerning health and well-being that all respondents were asked, irrespectively of their labour market status. These 47 questions are on the first 8 pages of the questionnaire. The questions have between three and six response categories and are primarily from the Stress Profile[14] and the SF-36 questionnaire[15]. The order of the questions was the same as in additional file 1: Table A.

The study has been notified to and registered by the Danish Data Protection Agency (Datatilsynet). According to Danish law, questionnaire based studies do not need approval by ethical and scientific committees, nor informed consent.

### Protocol

The original protocol for the survey did not specify any objectives or hypotheses (or primary and secondary outcome measures) concerning the effect of mode of data collection. We thus tested the hypotheses that we identified in the literature and listed in the introduction, and we estimated any differences. Questions concerning loneliness, well-being, self-esteem, unhappiness, nervousness, worry, depression, or trouble sleeping are mentioned in the literature as sensitive[1,7,16]. We categorised our 47 items as belonging under one of the four mentioned self assessments: well-being, self-esteem, depression or the additional heading stress, or under one of two behaviours: smoking habits and use of medicine. Thus all 47 questions are either sensitive or concerning socially undesirable behaviour. The categorization appears from the additional file 1: Table A (cf. the "Theme" column) and 2 and the analyses are presented accordingly. The distinction between self assessments and behaviours were added post-hoc.

### Statistical analysis

We used the chi-square test to test the hypothesis of telephone interviews resulting in higher response rates than mailed questionnaires (Hypothesis 1) and logistic regression to assess differential bias between mode of data collection, age and gender; marginal associations between mode of data administration and demographic data were examined using the chi-square test of independence.

We tested the hypotheses of higher rates of missing responses among questionnaire respondents than among telephone respondents (Hypothesis 2) and of greater willingness to answer sensitive questions in questionnaires than in telephone interviews (Hypothesis 3) by Fishers exact test (two-sided for all items since both hypotheses are relevant for all items).

We used the chi-square test to test whether telephone respondents had a preference for extreme (i.e. the highest possible and the lowest possible) response categories compared to questionnaire respondents (Hypothesis 4) and whether the level of reported health and well-being differed according to mode of data collection (Hypothesis 5) (The null hypotheses of these hypotheses are the same, while the alternative hypotheses differ). The tendency to use the extreme response categories among phone respondents relative to mail respondents was quantified by the odds ratio for extreme replies versus the pooled middle categories, and the tendency to reply more positively was quantified by the odds ratio estimated in the proportional odds model. Responses in the "don't know" category were treated as missing values.

The same statistical tests are performed on 47 items so the traditional 5% level of statistical significance is replaced by a 0.1% level, i.e. a Bonferroni correction of the 5% level[17].

## Results

### Participant flow and follow up

Participants were sampled from the CRS early August 1997 and randomised to mode of data collection August 18^th^. Questionnaires were sent out around October 1^st ^and telephone interviews conducted concurrently until January 1998. Data on mode of data collection, gender and year of birth were available for the entire sample and indicated a successful randomization (Table 1). The flowchart indicates reasons for non-response.

**Table 1:** Marginal distribution of gender and age by allocation to mode of data collection

	Mailed questionnaire		Telephone interview	
	%	N	%	N

Gender				
Men	50.5	1319	49.0	681
Women	49.5	1292	51.0	708
				
Age				
20 – 30	26.7	698	25.3	352
31 – 40	25.0	653	25.0	347
41 – 50	24.9	649	25.3	351
51 – 60	23.4	611	24.4	339
				
Total	100	2611	100	1389

### Response rates

Overall, the response rates for the two modes of data collection were similar; 58.1% among persons randomly assigned to questionnaires and 56.2% among persons randomly assigned to telephone interviews (p = 0.26). However, some age and gender differences were hidden (Table 2). Women and persons in their thirties were more willing to respond to the mailed questionnaire. A logistic regression analysis confirmed these interactions between mode of data collection and respectively gender (p = 0.001) and age (p = 0.01) (Estimates not shown). The specific difference for women in their thirties (69% versus 50%) was even larger than shown in Table 2. Only persons responding by the mode to which they were randomised, were included in these analyses; cross-over respondents were treated as non-respondents.

**Table 2:** Response rates by gender and age

	Mailed questionnaire		Telephone interview	
	%	N	%	N

Total	58.1	2611	56.2*	1389
				
Gender				
Men	54.2	1319	57.9	681
Women	62.0	1292	54.7	708
				
Age				
20 – 30	56.6	698	56.3	352
31 – 40	62.8	653	51.6	347
41 – 50	59.0	649	61.8	351
51 – 60	53.7	611	55.2	339

*153 persons assigned to telephone interviews answered mailed questionnaires. The response rate for the combined data collection strategy was 67.2%.

Self reported demographic data were compared for the two types of respondents. No differences were found in the marginal distribution of schooling (p = 0.13), labour market status (p = 0.43), number of children living at home (p = 0.20), or number of children under the age of six living at home (p = 0.77). Significant differences were found for vocational training (p = 0.004) and cohabitation (p = 0.001). Among telephone respondents, there was a larger percentage of persons with a further and higher education than among mail respondents (11.8% versus 7.3%) and a larger percentage of persons who had always lived alone (9.1% versus 5.3%).

### Missing

For all items there were more missing values among questionnaire respondents than among telephone respondents (Table 3). For smoking habits the difference was non-significant; for most of the self assessment questions and for use of medicine the difference was significant (Additional file 1: Table A). The lowest rate of missing replies (around 1%) in the mailed questionnaire is consistently achieved for stand-alone-questions (e.g. smoking habits), an intermediate rate (around 2%) is consistently achieved for the first question in blocks of questions with common introduction, and the poorest rate (3 – 6%) for the remaining questions.

**Table 3:** Range of percentages of missing values

Theme	Number of questions	Range of missing* (%)	
		
		Telephone	Mail
Medicine	3	0	1.58 – 3.63
Smoking habits	1	0	0.73
Self-esteem	9	0 – 0.51	1.85 – 6.00
Well-being	11	0 – 0.13	0.92 – 6.00
Depression	7	0 – 0.13	2.04 – 4.88
Stress	15	0 – 0.13	2.04 – 4.75

* Percentage of respondents not having answered a particular item.

### The "don't know" option

The "don't know" category was used much more frequently among questionnaire respondents (Table 4). Being the middle response category, the "don't know" option might also be regarded as a neutral response, at least in the mailed questionnaire.

**Table 4:** Percentage of respondents answering "don't know"

Item	Don't-knows (%)	
	
	Telephone	Mail
I seem to get sick a little easier than other people	3.33	10.22
I am as healthy as anybody I know	2.69	13.90
I expect my health to get worse	4.36	23.76
My health is excellent	2.05	10.39

### Response pattern

For the health behaviours we found no significant differences in the response patterns (Table 5).

**Table 5:** Odds ratios for more extreme responses in telephone interviews

Theme	Number of mode-sensitive* items of total number of items	Median (and maximum)odds ratio among mode-sensitive items
*Health behaviour*		
Medicine	0/3	-
Smoking habits	0/1	-
*Self assessments*		
Self-esteem	8/9	1.57 (2.57)
Well-being	9/11	1.55 (2.20)
Depression	3/6	2.12 (2.21)
Stress	12/15	1.67 (2.44)

* Items where the hypothesis of identical distributions among telephone respondents and respondents to mailed questionnaires was rejected.

For the 41 health self assessment items the response pattern was statistically significant different for 32 items (Additional file 2: Table B). All significant differences were in the same direction with regard to extreme responses (Hypothesis 4): more use of the extreme responses in telephone interviews. The median estimated odds ratio for the 32 items was 1.67 for more extreme responses in telephone interviews. The median estimated odds ratios within each theme were in the range 1.55 – 2.12 and the largest estimated odds ratios were in the range 2.20 – 2.57 (Table 5). Odds ratios above 1.2 were estimated for the six 6 "depression" and "stress" items that did not differ significantly between the modes. The pattern was slightly more complex regarding the tendency for positive responses (Hypothesis 5): of the 32 significantly different response patterns, 26 were shifted toward a more positive reporting among the telephone respondents (Additional file 2: Table B). The median estimated odds ratio for the 26 items was 1.73 for more positive responses in telephone interviews. The median odds ratios (within each theme) were in the range 1.60 – 1.99 and the largest odds ratios were in the range 1.82 – 2.97 (Table 6). Odds ratios above 1.2 were estimated for the six 6 "depression" and "stress" items that did not differ significantly between the modes. Of the six items shifted in the opposite direction, four were grouped under the self-esteem heading.

**Table 6:** Odds ratios for more positive responses in telephone interviews

Theme	Number of mode-sensitive* items	Median (and maximum) odds ratio among mode-sensitive items	Number of mode-sensitive items	Median (and minimum^‡^) odds ratio among mode-sensitive items
	
	with OR^†^>1		with OR<1	
*Health behaviour*				
Medicine	0	-	0	-
Smoking	0		0	-
*Self assessments*				
Self-esteem	4	1.99 (2.97)	4	0.88 (0.70)
Well-being	8	1.60 (2.01)	1	(0.75)
Depression	3	1.74 (1.82)	0	-
Stress	11	1.64 (2.27)	1	(0.73)

* Items where the hypothesis of identical distributions among telephone respondents and respondents to mailed questionnaires was rejected.

^† ^OR, odds ratio.

^‡ ^Since OR<1

Adjustment for the observed differences in gender and age response rates did not alter the magnitude of the estimates appreciably (Data not shown).

If the "don't know" category was analysed as the neutral middle response, the estimates were increased and the conclusion was strengthened (Data not shown).

## Discussion

### Findings

No differences were found in the overall response rate but a breakdown on age and gender showed that women and persons in their thirties had a higher response rate to the mailed questionnaire compared to the telephone survey. The rate of missing responses is clearly higher among questionnaire respondents than among telephone respondents. In relation to questions on behaviour, no difference between the two modes of data collection was found in the use of extreme response categories and no shift in the level was found between the modes. In relation to questions involving self assessments, telephone respondents had a strong preference for extreme response categories compared to questionnaire respondents and most items were shifted towards a more positive reporting among the telephone respondents.

When it comes to utilizing extreme response categories and to shift in level of response, the odds ratio estimates were above 2 for several items, i.e. as large as many effects studied in epidemiology.

### Strengths and weaknesses of our study

The unique opportunity in the presented data set to study mode sensitivity on a range of questions has so far been unused. The difficulties we met trying, retrospectively, to construct a flowchart and to verify the coding of variables emphasise the importance of completing these tasks concurrently with conducting the study[18]. These deviations from the ideal, however, do not seem to cause bias in the conclusions of our study. Our categorisation of individual items under the themes mentioned in the literature as sensitive might have been done differently by other researchers. However, we have preferred to maintain our pre-analysis categorisation. We do not suspect this categorisation to have any major influence on our results.

To counteract the multiplicity of analyses and outcomes we applied a conservative Bonferroni correction of the p-values. Nevertheless, the vast majority of statistical tests were significant and the emerging pattern of differences was fairly consistent.

The sample was a stratified sample of an adult population. The results are thus fairly generalizable compared to samples of more specific subgroups. However, the analysed questions concerned only health behaviour and self assessment items so generalization to other types of questions should be done cautiously.

### Comparison with other studies

Compared to most previous studies, our randomized design is strong.

It has previously been reported both that mailed questionnaires resulted in a lower[2,6,8], a higher[4], or the same[7] response rate as telephone interviews. The response rate will probably depend highly on the specific strategy and persistence in each trial arm[19]; the telephone response rate may also dependent on administrative accessibility of phone numbers and changes in phone technology.

The highly significant difference[4,7-9] and the missing rate of almost nil among the telephone respondents[4,8] are in accordance with other findings.

The cognitive demands on telephone respondents are higher than the cognitive demands on questionnaire respondents. Questionnaires enable the respondent to control both the pace and the order of interview[7]. A tendency for telephone respondents to prefer extreme response categories has also been reported in other studies[7,8] that primarily covered self assessments.

Due to the few missing responses among telephone respondents, the hypothesis of greater willingness to answer sensitive questions and questions concerning socially undesirable behaviour in questionnaires than in telephone interviews could not be confirmed by our data.

### Possible mechanisms

A majority of the questions considered here are sensitive according to the literature, and if interviewers are instructed not to accept item refusals it may be at the cost of sensitive questions being answered randomly or perhaps overly positive.

As already mentioned, most of the self assessment items were shifted towards a more positive reporting among the telephone respondents. However, the pattern was slightly more complex, since six of the mode-sensitive items shifted in the opposite direction. These six items all appear in blocks of questions formulated as statements with response categories indicating how well the statement fits the respondent's situation ("Correct", "Almost correct", "Somewhat correct", "Only slightly correct", "Incorrect"). Some statements are positive (E.g. "I feel I understand most of what is going on in my everyday life") and some are phrased using a negation (E.g. "So far, I have not had any clear direction or purpose in life"); five of the deviating items included negations and four of these concerned self-esteem. In Danish, the questions concerning self-esteem are strikingly longer and more long-windedly phrased than the rest of the questions. The long-windedness, the mix of positive statements and statements using negations, or both might explain the deviation from the overall pattern. However, these are post-hoc speculations.

According to the theory of response order effects, a preference for the first response categories is to be expected in visually presented questionnaires whereas a preference for the last mentioned response categories is to be expected in telephone interviews[20]. A shift in response pattern supporting this theory was observed for half of the items. (This theory coincides with the hypothesis of a shift toward a more positive reporting among the telephone respondents for questions using a negation and coincides with the hypothesis of a shift toward a more negative reporting among telephone respondents for questions positively phrased). Thus the mechanism of more positive reporting among the telephone respondents is stronger in our study than the proposed response order effect.

### Future research

In our study response patterns were similar for questions on health behaviour regardless of mode of data collection; whereas, response patterns were significantly different for questions on health self assessments. This finding was not one of our pre-specified hypotheses so an independent test of our finding would be welcome. Also similar tests and quantifications of mode sensitivity in a wider set of item types, e.g. items unrelated to health issues and items including opinions and values would be useful.

Costs were not booked in our study but ought to be booked in future studies. It is important additional information in decision making when comparing the two data collection methods in relation to differences in response rates, patterns of missing responses and response patterns and when optimizing reminder procedures. Calibration of the measurements from the two modes of data collection is also an important issue. This has to some extent been dealt with elsewhere[21].

## Conclusion

The mode of data collection affects the reporting of self assessed health items substantially. In epidemiological studies, the method effect may be as large as the effects under investigation. Caution is needed when comparing prevalences across surveys or when studying time trends.

## Competing interests

The author(s) declare that they have no competing interests.

## Authors' contributions

AH provided information on material and methods, HF re-established and analysed the data and wrote the first draft of the paper; OO and HF discussed and rewrote the draft several times, AH critically revised several of the drafts and agreed on a final version with OO and HF. All authors read and approved the final manuscript.

**Figure 1:**
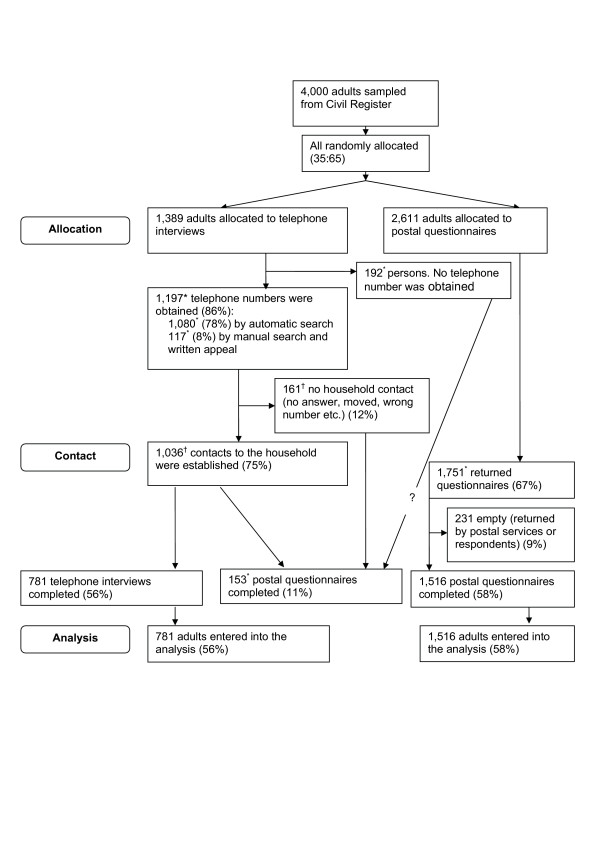
**Flow diagram**. The flowchart was not made immediately following data collection. Various slightly inconsistent summaries of the events between the random allocation and data processing exist. We have done our best to reconstruct the flowchart but had to indicate some unexplainable deviations between different archived summaries. Other versions of the flowchart may be requested from the authors. * Number deviates with 1–10 in different summaries of the data collection log. ^† ^Number deviates with more than 10 in different summaries of the data collection log.

## Pre-publication history

The pre-publication history for this paper can be accessed here:



## Supplementary Material

Additional File 1 Table A. PDF file containing a table of the rates of missing responses for all 47 items. Click here for file

Additional File 2 Table B. PDF file containing a table of estimated odds ratios for extreme responses and for more positive responses for all 45 items. Click here for file
