# Supplementary material for: A randomized trial of mailed questionnaires versus telephone interviews: Response patterns in a survey
Source: BMC Med Res Methodol. 2007 Jun 26;7:27. doi: 10.1186/1471-2288-7-27 (PMC1925106; doi:10.1186/1471-2288-7-27)
Supplement: Additional File 1 — Table A. PDF file containing a table of the rates of missing responses for all 47 items. [file 1471-2288-7-27-S1.pdf]

**Table A - Missing responses among respondents. Fisher's exact test (two-sided)**

| Item | Theme | Missing (%) | | p-value |
|---|---|---|---|---|
| | | Telephone | Mail | |
| Health in general | Well-being | 0 | 0.92 | 0.004 |
| Health compared to one year ago | Well-being | 0 | 1.12 | 0.001 |
| Been withdrawn | Depression | 0.13 | 2.04 | <0.0001 |
| Not able to deal with other people | Depression | 0.13 | 2.51 | <0.0001 |
| Not had the time to relax or enjoy myself | Well-being | 0 | 3.23 | <0.0001 |
| Found it difficult to be happy | Depression | 0 | 2.70 | <0.0001 |
| Have eaten for comfort | Depression | 0 | 3.76 | <0.0001 |
| Have been a bit touchy | Stress | 0 | 3.76 | <0.0001 |
| Have lacked initiative | Stress | 0 | 4.29 | <0.0001 |
| Have felt harassed | Stress | 0.13 | 4.49 | <0.0001 |
| Feeling full of pep | Stress | 0 | 2.04 | <0.0001 |
| A very nervous person | Stress | 0 | 2.97 | <0.0001 |
| Nothing could cheer me up | Depression | 0 | 3.36 | <0.0001 |
| Calm and peaceful | Well-being | 0.13 | 3.89 | <0.0001 |
| Having a lot of energy | Well-being | 0.13 | 4.49 | <0.0001 |
| Downhearted and blue | Depression | 0.13 | 4.88 | <0.0001 |
| Worn out* | Depression | 0 | 4.29 | <0.0001 |
| A happy person | Well-being | 0 | 5.41 | <0.0001 |
| Tired | Well-being | 0 | 4.49 | <0.0001 |
| Sick a little easier than other people | Well-being | 0 | 1.85 | <0.0001 |
| As healthy as anybody I know | Well-being | 0.13 | 5.08 | <0.0001 |
| Expect health to get worse | Well-being | 0.13 | 4.22 | <0.0001 |
| Excellent health | Well-being | 0 | 6.00 | <0.0001 |
| Painkillers* | Medicine | 0 | 1.58 | <0.0001 |
| Sedatives | Medicine | 0 | 3.50 | <0.0001 |
| Sleeping medicine | Medicine | 0 | 3.63 | <0.0001 |
| Have had stomach ache or problem* | Stress | - | - | - |
| Have had a tight chest or chest pains | Stress | 0 | 3.10 | <0.0001 |
| Have had palpitations | Stress | 0 | 3.17 | <0.0001 |
| Have been short of breath | Stress | 0 | 4.62 | <0.0001 |
| Have been dizzy | Stress | 0 | 4.29 | <0.0001 |
| Have had tension in various muscles | Stress | 0 | 3.83 | <0.0001 |
| Have had a tendency to sweat | Stress | 0 | 3.63 | <0.0001 |
| Have had problems concentrating | Stress | 0.13 | 4.75 | <0.0001 |
| Have had difficulty in taking decisions | Stress | 0 | 4.02 | <0.0001 |
| Have had difficulty with remembering | Stress | 0.13 | 4.68 | <0.0001 |
| Have found it difficult to think clearly | Stress | 0 | 3.83 | <0.0001 |
| Smoking daily | Smoking habits | 0 | 0.73 | 0.02 |
| I can cope with most situations in life | Self-esteem | 0.13 | 1.85 | 0.0001 |
| No clear direction or purpose in life | Self-esteem | 0.26 | 3.89 | <0.0001 |
| Not able to influence my future | Self-esteem | 0.13 | 4.68 | <0.0001 |
| What I do in my daily life is meaningful | Self-esteem | 0 | 5.74 | <0.0001 |
| Things happen that I do not understand | Self-esteem | 0.26 | 4.95 | <0.0001 |
| I have a great deal to live for | Self-esteem | 0.13 | 6.00 | <0.0001 |
| Know what I ought to do, but not able to | Self-esteem | 0.51 | 4.42 | <0.0001 |

| | | | | |
|---|---|---|---|---|
| Difficult to see how pieces in life connect | Self-esteem | 0.13 | 4.88 | <0.0001 |
| Understand most of my everyday life | Self-esteem | 0 | 4.95 | <0.0001 |

Solid horizontal lines indicate blocks of questions with a common introduction

* Three variables were partly corrupted during early data processing; two are left out from all further analyses; for "painkillers" see footnote to additional file 1: Table B.
