# Supplementary material for: A randomized trial of mailed questionnaires versus telephone interviews: Response patterns in a survey
Source: BMC Med Res Methodol. 2007 Jun 26;7:27. doi: 10.1186/1471-2288-7-27 (PMC1925106; doi:10.1186/1471-2288-7-27)
Supplement: Additional File 2 — Table B. PDF file containing a table of estimated odds ratios for extreme responses and for more positive responses for all 45 items. [file 1471-2288-7-27-S2.pdf]

**Table B - Estimated odds ratios for extreme responses and for more positive responses among telephone respondents**

| Item | Estimated odds ratios | | p-value[§] |
|---|---|---|---|
| | Extreme responses | More positive reporting | |
| *Medicine* | | | |
| Painkillers[*‡] | 0.97 | 0.89 | 0.16 |
| Sedatives[*] | 1.67 | 1.18 | 0.50 |
| Sleeping medicine[*] | 0.87 | 1.05 | 0.77 |
| *Smoking habits* | | | |
| Smoking daily[*] | 1.12 | 0.97 | 0.54 |
| *Self-esteem* | | | |
| I can cope with most situations in life | 1.97 | 1.92 | <0.0001 |
| No clear direction or purpose in life[*] | 1.05 | 0.87 | <0.0001 |
| Not able to influence my future[*] | 1.18 | 0.88 | <0.0001 |
| What I do in my daily life is meaningful | 2.57 | 2.97 | <0.0001 |
| Things happen that I do not understand[*] | 1.07 | 0.70 | <0.0001 |
| I have a great deal to live for | 2.00 | 2.05 | <0.0001 |
| Know what I ought to do, but not able to[*] | 1.26 | 0.98 | <0.0001 |
| Difficult to see how pieces in life connect[*] | 0.96 | 0.94 | 0.006 |
| Understand most of my everyday life | 1.88 | 1.90 | <0.0001 |
| *Well-being* | | | |
| Health in general | 1.76 | 1.39 | <0.0001 |
| Health compared to one year ago | 0.93 | 0.99 | 0.90 |
| Sick a little easier than other people[*†] | 0.81 | 0.79 | 0.13 |
| As healthy as anybody I know[†] | 1.48 | 1.54 | <0.0001 |
| Expect health to get worse[*†] | 1.55 | 1.63 | <0.0001 |
| Excellent health[†] | 1.83 | 1.79 | <0.0001 |
| Not had the time to relax or enjoy myself[*] | 1.13 | 0.75 | <0.0001 |
| Calm and peaceful | 1.52 | 1.56 | <0.0001 |
| Having a lot of energy | 1.24 | 1.76 | <0.0001 |
| A happy person | 1.79 | 2.01 | <0.0001 |
| Tired[*] | 2.20 | 1.31 | <0.0001 |
| *Depression* | | | |
| Been withdrawn[*] | 1.64 | 1.45 | <0.0001 |
| Not able to deal with other people[*] | 2.12 | 1.74 | <0.0001 |
| Found it difficult to be happy[*] | 2.21 | 1.82 | <0.0001 |
| Have eaten for comfort[*] | 1.57 | 1.52 | 0.002 |
| Nothing could cheer me up[*] | 1.42 | 1.41 | 0.005 |
| Downhearted and blue[*] | 1.33 | 1.21 | 0.01 |
| *Stress* | | | |
| Have been a bit touchy[*] | 1.53 | 1.08 | <0.0001 |
| Have lacked initiative[*] | 2.18 | 1.82 | <0.0001 |
| Have felt harassed[*] | 1.11 | 0.73 | <0.0001 |
| Feeling full of pep | 1.31 | 1.72 | <0.0001 |
| A very nervous person[*] | 1.66 | 1.53 | <0.0001 |
| Have had a tight chest or chest pains[*] | 1.47 | 1.44 | 0.006 |
| Have had palpitations[*] | 1.64 | 1.60 | 0.0001 |

| | | | |
|---|---|---|---|
| Have been short of breath* | 2.25 | 2.01 | <0.0001 |
| Have been dizzy* | 1.94 | 1.91 | <0.0001 |
| Have had tension in various muscles* | 1.67 | 1.21 | <0.0001 |
| Have had a tendency to sweat* | 2.44 | 2.27 | <0.0001 |
| Have had problems concentrating* | 1.73 | 1.64 | <0.0001 |
| Have had difficulty in taking decisions* | 1.58 | 1.51 | <0.0001 |
| Have had difficulty with remembering* | 1.28 | 1.26 | 0.05 |
| Have found it difficult to think clearly* | 1.36 | 1.40 | 0.002 |

* Questions were reversed (either by negation or by using negatively loaded terms)

† "Don't know" responses were treated as missing values

‡ Variable partly corrupted; the middle categories "one or several times a week" and "one or several times a month" were combined in the analyses.

§ Chi-square test for identical distributions on the complete scale
